# Unusual N Gene Dropout and Ct Value Shift in Commercial Multiplex PCR Assays Caused by Mutated SARS-CoV-2 Strain

**DOI:** 10.3390/diagnostics12040973

**Published:** 2022-04-13

**Authors:** Petros Bozidis, Eleni T. Tsaousi, Charilaos Kostoulas, Prodromos Sakaloglou, Athanasia Gouni, Despoina Koumpouli, Hercules Sakkas, Ioannis Georgiou, Konstantina Gartzonika

**Affiliations:** 1 Department of Microbiology, Faculty of Medicine, School of Health Sciences, University of Ioannina, 45110 Ioannina, Greece; stm02754@uoi.gr (E.T.T.); p.sakaloglou@uoi.gr (P.S.); kgartzon@uoi.gr (K.G.); 2 Laboratory of Medical Genetics in Clinical Practice, Faculty of Medicine, School of Health Sciences, University of Ioannina, 45110 Ioannina, Greece; chkost@uoi.gr (C.K.); igeorgio@uoi.gr (I.G.); 3 Department of Microbiology, University Hospital of Ioannina, 45500 Ioannina, Greece; agouni@uhi.gr (A.G.); dkoumpouli@uhi.gr (D.K.)

**Keywords:** SARS-CoV-2, variant, diagnostic quantitative RT-PCR, B.1.1.318

## Abstract

Several SARS-CoV-2 variants have emerged and early detection for monitoring their prevalence is crucial. Many identification strategies have been implemented in cases where sequencing data for confirmation is pending or not available. The presence of B.1.1.318 among prevalent variants was indicated by an unusual amplification pattern in various RT-qPCR commercial assays. Positive samples for SARS-CoV-2, as determined using the Allplex SARS-CoV-2 Assay, the Viasure SARS-CoV-2 Real Time Detection Kit and the GeneFinder COVID-19 Plus RealAmp Kit, presented a delay or failure in the amplification of the N gene, which was further investigated. Whole-genome sequencing was used for variant characterization. The differences between the mean Ct values for amplification of the N gene vs. other genes were calculated for each detection system and found to be at least 14 cycles. Sequencing by WGS revealed that all the N gene dropout samples contained the B.1.1.318 variant. All the isolates harbored three non-synonymous mutations in the N gene, which resulted in four amino acid changes (R203K, G204R, A208G, Met234I). Although caution should be taken when the identification of SARS-CoV-2 variants is based on viral gene amplification failure, such patterns could serve as a basis for rapid and cost-effective screening, functioning as indicators of community circulation of specific variants, requiring subsequent verification via sequencing.

## 1. Introduction

Since its beginning, the SARS-CoV-2 pandemic has been a challenge for molecular diagnostics. As the gold standard for molecular detection of SARS-CoV-2 infection is RT-qPCR [1], various commercial RT-qPCR kits have been developed for this purpose (https://www.finddx.org/test-directory/. Accessed 13 February 2022). Most target SARS-CoV-2 genes, such as RNA-dependent RNA polymerase (RdRp), the envelope (E), spike (S) and nucleocapsid (N), or viral genomic regions such as ORF1a/b and ORF8 [2,3]. 

SARS-CoV-2 is a positive-stranded RNA virus that, similarly to other RNA viruses, exhibits rapid evolution, mainly due to two mechanisms: (a) incorporation of random mutations caused by the high error rates (1/34,000) of the viral polymerase RdRp and (b) homologous and heterologous recombination via strand switching by the same enzyme during viral replication [4]. Thus, there are often cases of a new mutant strain, for which the RT-qPCR fails to amplify one of the selected viral targets while the rest are detected. This phenomenon is called dropout and has been previously associated with amplicons generated from either the S or N gene [5,6,7,8,9,10,11,12]. To deal with the problem of the amplification failure, most of the available RT-qPCR protocols for SARS-CoV-2 target at least two different regions in the viral genome [13,14].

Although the performance of certain commercial assays may be affected by the emergence of new mutations, the change in the amplification pattern may help with the early identification of new variants. Here, we report an approach that allows for presumptive characterization of the B.1.1.318 lineage by using the pattern of amplification of three different widely used commercial RT-qPCR tests for SARS-CoV-2 detection.

## 2. Materials and Methods

### 2.1. Samples and RT-qPCR

Nasopharyngeal swabs from suspected cases were collected from April to June of 2021 and routinely tested for the presence of SARS-CoV-2 immediately upon arrival at the Laboratory of Microbiology of the University Hospital of Ioannina, Greece. Total RNA was extracted from patient samples using the KingFisherTM Flex Purification System (Life Technologies Holdings Pte. Ltd., Singapore) according to the manufacturer’s guidelines.

The extracted RNAs were tested for the SARS-CoV-2 genome using three widely used commercial multiplex RT-qPCR assays: the Allplex SARS-CoV-2 Assay (Seegene Inc., Seoul, Korea), which targets the E, N and RdRp/S genes; the Viasure SARS-CoV-2 Real Time Detection Kit (CerTest Biotec, Zaragoza, Spain), which targets the Orf1a/b region and the N gene; and finally, the GeneFinder COVID-19 Plus RealAmp Kit (Osang Healthcare Co., Ltd., Anyang-si, Korea), which targets the E, N and RdRp genes. All RT-qPCR assays include an internal control for verification of the nucleic acid extraction procedure and identify possible PCR inhibition. The amplification reactions were performed in a single tube according to the manufacturer’s protocols, either on a CFX96 Touch Real-Time PCR Detection System (Bio-Rad Laboratories, Inc., Hercules, CA, USA), or on an ELITE InGenius system (ELITechGroup Molecular Diagnostics) in the case of the GeneFinder COVID-19 Plus RealAmp Kit. Following assay completion, Ct values were calculated either automatically (using Seegene Viewer ver3.24 or ELITE InGenius curve analysis software) or manually (based on Viasure SARS-CoV-2 Real Time Detection Kit instructions).

### 2.2. Whole-Genome Sequencing

RNA samples with and without the dropout effect were reverse transcribed and sequenced by whole-genome sequencing (WGS). Τwo primer pools were utilized to amplify the whole viral genome into 400 bp amplicons, using two different multiplex PCR experiments for each sample (QIAseq SARS-CoV-2 Primer Panel Kit). Sequencing libraries were constructed using the QIAseq FX DNA library Kit and quantified with the KAPA Library Quantification Kit (KAPA). Libraries were sequenced on an Illumina MiSeq system using a Reagent Kit v2 (Illumina, San Diego, CA, USA). The viral consensus sequences were assembled by mapping them to the reference sequence of SARS-CoV-2 (mn908947/nc_045512) using the CLC Genomics Workbench v21.0.5 (Qiagen GmbH, Hilden, Germany). Each variant was confirmed in the IGV (Integrated Genome Viewer) and lineages were assigned using the pangolin tool (https://cov-lineages.org/. Accessed 9 September 2021).

### 2.3. Statistical Analysis

The mean Ct values for all genes are presented as the mean ± standard deviation. The differences between the mean Ct values of the N gene vs. the E or RdRp/S genes (Allplex SARS-CoV-2) and the N gene vs. the Orf1ab region (Viasure SARS-CoV-2 Real Time Detection) were calculated from each group of samples, with and without dropout, and tested for significance with an independent-group *t*-test (all *p* < 0.05).

## 3. Results

### 3.1. N Gene Dropout in RT-qPCR

**Figure 1:**
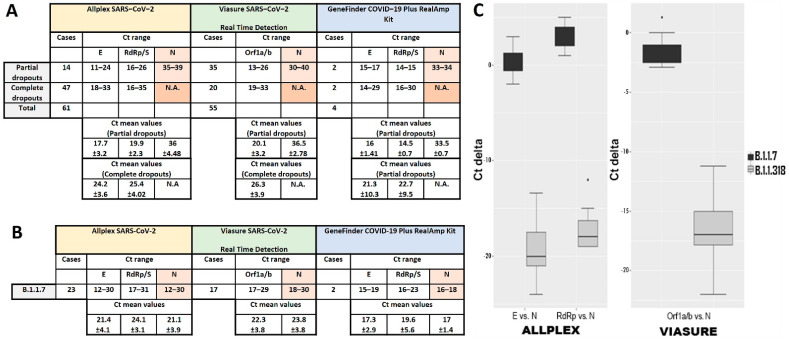
(**A**) All three commercial detection kits show N amplification delay (partial dropout) or failure (complete dropout) in samples infected by the B.1.1.318 strain. (**B**) 42 samples infected by the B.1.1.7 strain were tested with all three detection kits for comparison. RT-qPCR data as Ct range and Ct mean values for each target of each molecular assay are depicted. (**C**) Distribution of delta Ct values for cases without dropout infected with B.1.1.7 (black box plots) and with partial dropout infected with B.1.1.318 variant (grey box plots). The visualization of differences in the Ct values of N vs. E or RdRp/S genes in Allplex SARS-CoV-2 assay is presented in left graph and between N vs. Orf1a/b in Viasure SARS-CoV-2 Real Time Detection in right graph both for the groups of the B.1.1.7 and B.1.1.318 variants. The horizontal lines represent mean values of differences of Ct values.

**Figure 2:**
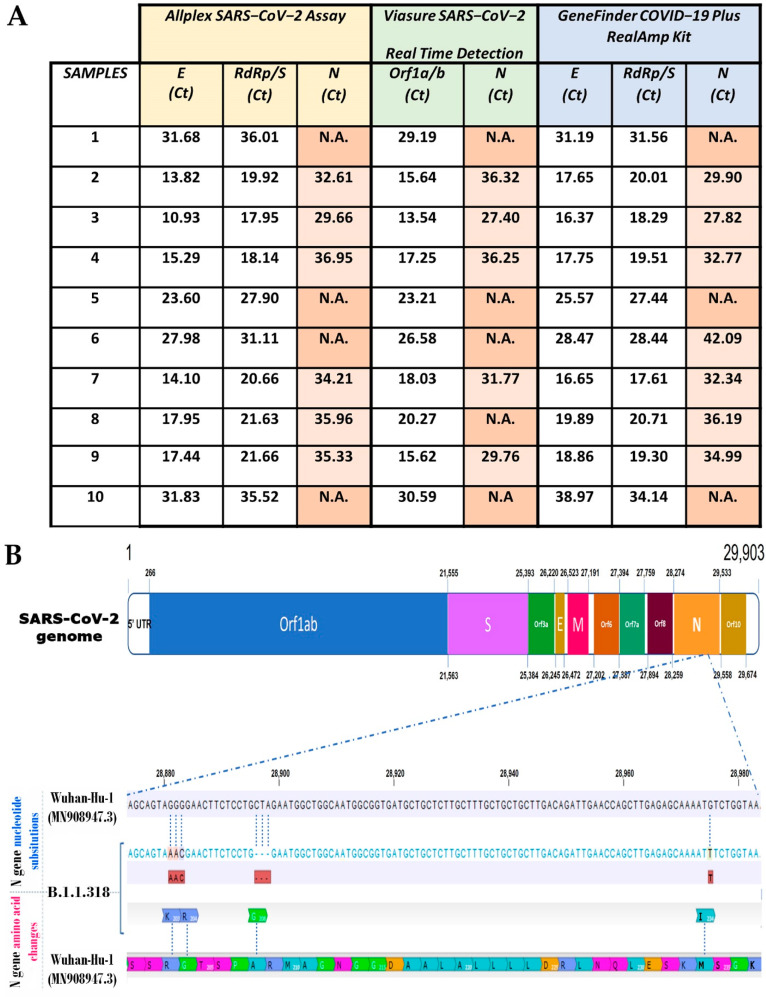
(**A**) The 10 random samples out of 120 that were infected with the B.1.1.318 strain showed similar N gene amplification delays or failures when tested with all three detection kits in parallel. (**B**) Mutations of the N gene sequence of B.1.1.318 isolates that affect primer–probe annealing.

During the study period, approximately 37,500 samples were examined for the presence of SARS-CoV-2 using three commercial RT-qPCR kits in rotation. Among the 1268 positive samples, an unusual delay or failure in the amplification of the N gene was observed in 120 (9.5%) of cases. The dropout was “partial” in 51 of these samples and “complete” in 69, meaning that the Ct value was either above or below (not applicable, N.A.) the threshold, respectively. In total, assays using the Allplex SARS-CoV-2 Assay, Viasure SARS-CoV-2 Real Time Detection Kit and GeneFinder COVID-19 Plus RealAmp Kit gave a Ct delay or failure for the N gene in 61, 55 and 4 out of 120 dropout samples, respectively (Figure 1A). As the dropout samples emerged through a random rotation of these detection kits, we wanted to find out if we could observe a similar Ct shift in each assay running the same samples. Therefore, we chose ten dropout samples both with partial or complete dropouts and assayed them, applying the three kits in parallel. The dropout effect for the N gene amplification, either partial or complete, was observed in all trials. In particular, the Allplex SARS-CoV-2had 6/10 partial dropouts (and 4 complete) with a mean Ct value for the N gene of 33.07 ± 3.3, the Viasure SARS-CoV-2 Real Time Detection had 5/10 partial dropouts (and 5 complete) with a mean Ct value of 34.12 ± 2.6 and, finally, the GeneFinder COVID-19 Plus RealAmp had 7/10 partial dropouts (and 3 complete) with a mean Ct value of 33.73 ± 4.6 (Figure 2A). Thus, not only was a delay in N gene amplification observed for the same samples in all cases, but the Ct shift was also similar for all three commercial detection kits.

### 3.2. Definition of N Gene Dropout

To determine the range of the Ct shift for the N gene and define the dropout effect in terms of Ct values, we compared the mean Ct values of the N gene against the mean Ct values of the other viral targets, and we observed an N delay in all assays of approximately 14 cycles at least (Figure 1A). In more detail, the Allplex SARS-CoV-2 gave a mean Ct value for the N gene of 19.46 ± 2.89 and 17.33 ± 2.02 cycles higher than the values for the E and RdRp/S genes, respectively, Viasure SARS-CoV-2 Real Time Detection gave a mean Ct value for the N gene of 16.4 ± 2.33 cycles higher than the Orf1a/b region (Figure 1C), while for GeneFinder COVID 19 Plus RealAmp, N gene amplification had delays of 17.5 ± 2.12 and 19 cycles compared to the amplification of the E and RdRp genes. Moreover, comparing the Ct values of viral targets other than the N gene, we realized that the difference between groups with partial and complete dropouts were dependent on the viral load of the samples. The viral load was higher in partial dropout samples than in complete dropout samples.

The extent of the N gene amplification delay became clear when compared to the Ct values for the N gene that are usually seen regardless of the molecular assay used. For comparison, 42 samples were used, which were found to belong to the B.1.1.7 lineage, as revealed by WGS. The B.1.1.7 variant was dominant in the community at that time. These samples were detected as positive for SARS-CoV-2 using the three kits during the same period that the dropout samples appeared. The mean Ct values for all viral targets of each kit are presented in Figure 1B. As can be observed, the mean Ct value for the N gene is similar to or 1–3 cycles higher or even lower than the mean Ct values of the rest of the viral targets (Figure 1C). Similar values have been observed in all samples examined so far, which included all known SARS-CoV-2 variants at the time (data not shown). Thus, the unusual pattern of the N gene Ct shift denotes the detection of a specific variant.

### 3.3. N Gene Amplification Delay Is Specific for the B.1.1.318 Variant

As the delay of the N gene in RT-qPCR had not previously been observed in other cases, we thought that the group of the 120 dropout samples might contain the same variant or variants with similar mutations that affect N gene amplification. Therefore, we sequenced all 120 samples and found that all samples contained only the B.1.1.318 variant. The N gene sequences of our isolates showed three nucleotide non-synonymous mutations compared to the reference strain of Wuhan (GenBank sequence accession: MN908947.3). These mutations include a substitution of three nucleotides (GGG to AAC) in position 28881–28883 nt, an in-frame deletion of three nucleotides (CTA) in position 28896–28898 nt and a single nucleotide polymorphism in position 28,975 of the viral genome (Figure 2B). These nucleotide substitutions result in four amino acid changes, which include a substitution of two amino acids at positions 203 and 204 (R203K, G204R), a deletion of two amino acids at position 208 and 209 and insertion of another without a frameshift (A208G) and one additional amino acid substitution at position 234 (M234I) (Figure 2B). Hence, the mutated N protein in the B.1.1.318 variant seems to have one less residue (418 aa) than the reference sequence for the N protein (QHD43423.2).

## 4. Discussion

Since the beginning of the COVID-19 pandemic, diagnostic laboratories around the world have been struggling to identify emerging SARS-CoV-2 variants in a rapid and accurate way [9,15]. Public health policies depend on knowledge of the circulation of such variants and tools for rapid identification upon their introduction into local communities. When sequencing capacity is limited, or early detection of SARS-CoV-2 variants is mandatory, alternative methods such as diagnostic screening with nucleic acid amplification technique (NAAT)-based methods should be applied [16].

A plethora of commercial NAAT kits are used for the detection of multiple viral loci and even some characteristic mutations of the S gene [17]. In most cases, the amplification of the viral amplicons follows the same pattern, which is reflected in the similarity in the Ct values for each gene. A different pattern could be observed when molecular diagnostic assays appear either delayed or to have completely failed in the amplification of one of the viral targets, due to the accumulation of mutations in the corresponding genes. These dropouts have been connected with amplification failure of either the S or the N gene and are indicative of the presence of certain variants [5,6,7,8,9,10,11,12]. Recently, the European CDC and WHO have noted a pattern of detection associated with S gene dropout that may help with early identification of the Omicron (B.1.1.529) variant, pending sequencing confirmation [16,18].

In the present study, both delays (high viral load) and complete failures (low viral load) of N gene amplification were observed specifically in samples that contain the B.1.1.318 variant. All RT-PCR-based results indicative of this variant were confirmed by sequencing. To our knowledge, this is the first study reporting either complete or partial N gene dropouts in a large number of samples in relation to the B.1.1.318 variant. In a similar publication, two cases belonging to the B.1.1.318 lineage showed no amplification signal for the N gene [19]. As determined by WHO, B.1.1.318 belongs to the group of variants under monitoring, carrying mutations that have been implicated in increased transmissibility or decreased vaccine efficacy [20].

N gene dropout has previously been reported as indicative for the B.1.1.7 variant, commonly known as the Alpha variant. The detection kits that failed to amplify the N gene in the B.1.1.7 variant were the TaqPath COVID-19 CE-IVD RT-PCR Kit manufactured by Thermo Fisher and the Allplex SARS-CoV-2/FluA/FluB/RSV Assay manufactured by Seegene (Table 1). These reports are in agreement with our results. Indeed, Wollschlaeger et al. reported that the Ν gene shift or N gene dropout caused by the B.1.1.7 variant can only be detected using Allplex SARS-CoV-2/FluA/FluB/RSV and not Allplex TM 2019-nCoV of the same company nor Viasure SARS-CoV-2 [11]. On the contrary, Lesbon et al. has recently reported the N gene amplification failure by qRT-PCR, using the GeneFinder COVID 19 Plus RealAmp kit, in samples infected with the P1 (or P.1.1) variant [21]. The researchers attribute the failure in amplification to three mutations in the N gene sequence, one of which (GGG to AAC) is the same as the one we detected in our own strains. We could not assess these findings and know how the GeneFinder would react against the P.1 variant detection since this variant had a very limited distribution among the Greek population (https://eody.gov.gr/wp-content/uploads/2022/03/covid-gr-daily-report-20220331.pdf. Accessed 7 April 2022).

Other cases where N gene delay has been observed in positive samples could be due to low viral loads. It is observed usually in the late stages of infection in samples from patients that are convalescing [22]. As the patients recover, there is gradually a failure to amplify the targets for these samples by RT-qPCR. The genes that remain detectable present very high Ct values, suggestive of low viral loads. In our experience, which is in agreement with what has been reported, the last gene to become undetectable is usually the N gene [23,24]. On the other hand, late (Ct values > 39) unspecific amplifications have been observed for the N gene due to dimer formation in the N2 primer–probe set (CDC 2019-nCov Real-Time RT-PCR) used, and also for the E gene when the Charité protocol is used [24,25,26]. These unspecific signals in the late cycles of amplification of specific viral genes may cause false-positive results in negative samples.

Sequencing analysis revealed four amino acid mutations (R203K, G204R, A208G, M234I) in the N protein sequence of all our B.1.1.318 isolates. A search with the outbreak.info tool (https://outbreak.info/situation-reports. Accessed 29 March 2022) showed that these mutations were not novel for the N gene sequence. According to the same tool, the frequency of the four amino acid substitutions, R203K, G204R, A208G and M234I, in 9.525.430 sequences available from the GISAID database (as of 29 March 2022, with collection days 2020-1-29, 2020-1-29, 2020-3-3, 2020-1-26 to 2022-3-29) was 44%, 43%, <0.5% and 2%, respectively. As far as the B.1.1.318 lineage and sub-lineages are concerned, all four mutations are found mostly in the AZ.2 (93.3%) and AZ.2.1 (98%) sub-lineages (as of 5 April 2022, with collection days 2021-2-5 to 2021-8-19). Notably, the AZ.2 sub-lineage was predominant in Greece during the study period (https://cov-lineages.org/lineage.html?lineage=B.1.1.318. Accessed 25 February 2022). The R203K and G204R amino acid changes have been found in several variants beside B.1.1.318 and are considered to contribute to increased infectivity and fitness and are related to a severe clinical outcome [27,28]. The M234I amino acid substitution has also occurred independently in several variants and, when it is coupled with the A376 substitution, may confer to carrying variants the ability to escape detection by commercial antigen tests [29,30]. As far as the A208G amino acid is concerned, it seems to be predominant in (but not unique to) the B.1.1.318 lineage and sub-lineages. This mutation is mapped at the third amino acid of a predicted in silico motif of the conserved N-myristoylation site GDAAL (206–210 aa) [31]. Although in positions 3 and 4, most, if not all, residues are allowed (https://prosite.expasy.org/PDOC00008. Accessed 7 April 2022), the impact of this substitution on the interaction of the N protein with host proteins and viral pathogenesis is yet unknown.

The three commercial kits used for routine diagnosis have detected almost all the known circulating variants in Europe, among which are the Alpha (B.1.1.7 lineage), Beta (B.1.351 lineage), Delta (B.1.617.2 lineage), Delta Plus (B.1.617.2/AY.1 sub-lineage) and Omicron (B.1.1.529 lineage) variants, which have never shown such an unusual pattern before (data not shown). Thus, as summarized in Table 1, some of the commercial RT-qPCR kits used for SARS-CoV-2 detection may serve as screening tools for the rapid and cost-effective presumptive identification of certain variants, based on viral gene amplification dropouts. However, caution should be taken when such mutant screening patterns are used [6,32], as in some cases, they have led to misdiagnosis of the correct variant type. This highlights the importance of sequencing a part of the samples under investigation to confirm the usefulness and validity of the Ct value shift for tracking SARS-CoV-2 variants.

**Table 1:** List of commercial kits used for presumptive identification of specific SARS-CoV-2 variants based on viral gene amplification delays or failures.

Variant	Gene	Detection Kit	References
B.1.1.7	S dropout or delay	TaqPath COVID-19 CE-IVD RT-PCR Kit	[6,7,9,10]
		Helix^®^ COVID-19 Test	[8]
	RdRp/S double curve or low amplification	Allplex SARS-CoV-2 Assay	[33]
	N dropout or delay	TaqPath COVID-19 CE-IVD RT-PCR Kit	[12]
		Allplex SARS-CoV-2/FluA/FluB/RSV Assay	[11,12]
B.1.258	S dropout or delay	TaqPath COVID-19 CE-IVD RT-PCR Kit	[6]
B.1.617.2	RdRp/S double curve or low amplification	Allplex SARS-CoV-2 Assay	[33]
B.1.177 (B.1.177.75)	N dropout	TaqPath COVID-19 CE-IVD RT-PCR Kit	[5]
B.1.1.529	S dropout	TaqPath COVID-19 Combo Kit	[34]
B.1.1.318	N dropout	Amplidiag COVID-19	[19]
		PerkinElmer SARS-CoV-2 Real-time RT-PCR Assay	[19]
	N dropout or delay	Allplex SARS-CoV-2	This work
		VIASURE SARS-CoV-2 Real Time Detection Kit	This work
		GeneFinder COVID-19 Plus RealAmp Kit	This work
P.1 (or P.1.1)	N dropout	GeneFinder COVID-19 Plus RealAmp Kit	[21]

## Data Availability

Not applicable.
